# Can antibiotic preference affect bleeding in percutaneous nephrolithotomy? Retrospective comparative study of two commonly used antibiotics

**DOI:** 10.12669/pjms.36.4.1977

**Published:** 2020

**Authors:** Ali Akkoc, Cemil Aydin, Murat Ucar, Murat Topcuoglu

**Affiliations:** 1 Ali Akkoc, Department of Urology, Faculty of Medicine, Alanya Alaaddin Keykubat University, Alanya, Turkey; 2 Cemil Aydin, Department of Urology, Faculty of Medicine, Hitit University, Corum, Turkey; 3 Murat Ucar, Department of Urology, Faculty of Medicine, Alanya Alaaddin Keykubat University, Alanya, Turkey; 4 Murat Topcuoglu, Department of Urology, Faculty of Medicine, Alanya Alaaddin Keykubat University, Alanya, Turkey

**Keywords:** Antibiotic, Bleeding, Hemorrhage, Kidney stones, Percutaneous nephrolithotomy

## Abstract

**Objective:**

Bleeding is one of the most common and alarming complication of percutaneous nephrolithotomy (PCNL). In this study, we aimed to compare the effects of ciprofloxacin and cefuroxime on the bleeding in PCNL procedures.

**Methods:**

The study was a retrospective analysis of 97 patients who underwent PCNL between February 2011 and June 2017. We just included the patients who had single tract lower pole PCNL for more objective evaluation of bleeding in the study. The patients were divided into two groups as ciprofloxacin group (Group-I, n:40) and cefuroxime group (Group-II, n:56) according to the type of antibiotic used in the operation. Patient age, gender, body mass index, stone size, preoperative INR, preoperative and postoperative platelet counts and difference, operative time, need for blood transfusion, postoperative fever, hospital stay, postoperative hemoglobin and hematocrit drop were analyzed.

**Results:**

There was no statistically significant difference in patients’ gender distribution, body mass index, preoperative INR, preoperative and postoperative platelet counts, preoperative and postoperative platelet difference, duration of operation, hospital stay, postoperative fever and need for postoperative blood transfusion between two antibiotic groups (p > 0.05). Mean patient age was 42,75±16,97 in Group-I and 35,54±14,71 in Group-II (p < 0.05). The mean stone size of Group-I and Group-II were 27,23±7,05 mm and 30,59±8,20, respectively (p < 0.05). The mean postoperative hemoglobin and hematocrit drop were significantly higher in Group-I than in Group-II. The mean hemoglobin drop was 1,73±0,95 for Group-I and 1,28±0,67 for Group-II (p < 0.05). The mean hematocrit drop was 5,17±2,76 for Group-I and 3,80±1,99 for Group-II (p < 0.05).

**Conclusion:**

On the basis of the results of the initial study, the antibiotic preference in patients undergoing surgery may be one of the bleeding factors during and after PCNL.

## INTRODUCTION

Since its introduction, percutaneous nephrolithotomy has aimed to provide effective treatment of large and complex kidney stones by achieving high stone free rates with acceptable complications. The American Urological Association and Endourology Society Surgical Management of Stones Guidelines recommends percutaneous nephrolithotomy as the first line treatment for intrarenal stones larger than 2 cm and lower pole stones larger than 1cm.^1^ Success rates for the kidney stones have been reported that exceed 90% with PCNL. However, it hosts a significant morbidity risk, with various series describing a complication rate of 20.5%.^2^ Complications associated with percutaneous nephrolithotomy are fever or hypothermia; sepsis; organ injuries such as pleura, colon and other abdominal organs; renal collecting system perforation and obstruction; loss of renal function; position related peripheral nerve injury; thromboembolic complications; bleeding and death. Intraoperative and postoperative bleeding is still one of the most common, significant and alarming complication of PCNL. Various studies have reported that blood transfusion rates between <1% and 55%,^3^ and the average hemoglobin drop ranging between 2.1 and 3.3.^4^

Diabetes mellitus, preoperative anemia, increased stone burden, method of dilatation, the number of access, surgical technique and experience, duration of surgery, history of previous renal surgery have been known as predictive factors of bleeding in PCNL procedures. In the literature, there is no study regarding relationship between antibiotics and bleeding in PCNL procedures.

This study aimed to compare the effects of ciprofloxacin and cefuroxime, which were two commonly used antibiotics in urologic surgeries, on the bleeding in PCNL procedures.

## METHODS

The study was a retrospective analysis of 97 patients who underwent PCNL by the same experienced surgical team between February 2011 and June 2017. The local institute ethics committee approved the study (Ref.No.: 18926-205-02 dated July 9, 2017). All patients were evaluated by medical history and physical examination. Prior to operation, blood glucose, urea, creatinine, coagulation profile, complete blood count, urinalysis and urinary culture were evaluated in each patient. Preoperative radiologic investigation consisted of kidney-ureter-bladder (KUB) plain film, intravenous pyelogram and non-contrast spiral tomography (CT) in all cases. The mean size of the stone was measured with the maximum diameter of stone size shown in the CT. All patients were duly informed about PCNL procedure and written consent was taken before surgery.

Exclusion criteria were bleeding diathesis; aspirin or anticoagulant agent usage; comorbidities such as diabetes mellitus, hypertension, renal dysfunction; anatomical kidney abnormalities such as pelvic kidney, horseshoe kidney, rotation anomalies; history of previous renal surgery; extracorporeal shock wave lithotripsy; multi-tract PCNL; preoperative severe hydronephrosis and solitary kidney. We just included the patients who were performed single tract lower pole PCNL for more objective evaluation of intraoperative and postoperative bleeding in the study. The patients were divided into two groups according to the type of antibiotic used in the operation.

Our standard antibiotic regimen for PCNL procedures was ciprofloxacin 400 mg IV or cefuroxime 1.5 g IV, every 12 hours, depending on availability in the hospital pharmacy. They were given from preoperative day to until the day of discharge and 60 minutes before the operation. Forty of the 96 patients who received PNCL indications were administered ciprofloxacin (Group-I) and 56 patients were given cefuroxime (Group-II). The general anesthesia protocol used in the operation was the same for all patients. It was induced with intravenous propofol (2 mg/kg) plus fentanyl (2 μg/kg), and neuromuscular blockade was achieved with rocuronium (0.6 mg/kg). After tracheal intubation, anesthesia was maintained with one minimum alveolar concentration (MAC) of sevoflurane (2%–2,5%) in a 50% oxygen and air. Remifentanil infusions (0.1–0.2 μg/kg/min) were used for analgesia. Preoperative, operative and postoperative parameters including patient age, gender, body mass index (BMI), stone size, preoperative INR, preoperative and postoperative platelet (plt) counts and difference, operative time, need for blood transfusion, postoperative fever, hospital stay, postoperative hemoglobin (Hb) and hematocrit (Hct) drop were analyzed. Operative time was calculated based on procedural start and stop times.

### Surgical technique

Under the general anesthesia using the same type of medication, retrograde ureteric catheterization with a 5 or 6 French (F) open-ended ureteric catheter was performed by using a rigid cystoscope or ureterorenoscope under C arm fluoroscopy. The patients were placed to the prone position. A radio-opaque fluid was administered through the ureteral catheter and the pelvicalyceal system was visualized via C arm fluoroscopy. After identifying the posterior lower pole calyx, renal access was achieved by using an 18 Gauge (G) percutaneous access needle. A 0.038” hydrophilic guide wire was inserted through the needle into the ureter or collecting system. Following dilatation of the tract using Amplatz renal dilatators, a 25 F nephroscope was inserted into the kidney through a 30 F percutaneous access sheath. Stone fragmentation was carried out with ballistic lithotripter. Forceps were used to remove stone fragments. At the end of the procedure, a 16f nephrostomy catheter was placed into the renal collecting system. When the patients were admitted to the urology service from operating theatre, complete blood count was routinely performed at the first postoperative hour.

### Statistical analysis

IBM SPSS Statistics 22.0 (IBM Corp. Released 2013, IBM SPSS Statistics for Windows, Version 22.0, IBM Corp.) program was used for statistical analysis. In the comparison of continuous variables between the groups, it was determined whether they were parametric or non-parametric by Shapiro Wilk test. Categorical characteristics were presented as numbers; continuous measurements were given as mean ± standard deviation and median-IQR. Chi square test was used to compare categorical variables. In the continuous variables, Student T test was used for parametric variables and Mann Whitney U test was used for nonparametric variables. A p value of <0.05 was considered statistically significant.

## RESULTS

Patients’ gender distribution, BMI, preoperative INR, preoperative Plt count, postoperative Plt count, preoperative and postoperative plt difference, duration of operation, hospital stay, postoperative fever and need for postoperative blood transfusion showed no significant difference between two antibiotic groups (p > 0.05). The patients consisted of 19 male patients and 21 female patients for Group-I, and 27 male patients and 29 female patients for Group-II (p=0.945). Mean patient age was significantly higher in Group-I than in Group-II, and they were 42,75±16,97 and 35,54±14,71, respectively (p < 0.05). Mean stone size for Group-I was significantly lower than Group-II. It was 27,23±7,05 mm for Group-I and 30,59±8,20 mm for Group-II (p < 0.05). Mean postoperative hemoglobin and hematocrit drop were significantly higher in Group-I than in Group-II. Mean hemoglobin drop was 1,73±0,95 for Group-I and 1,28±0,67 for Group-II (p < 0.05). Mean hematocrit drop was 5,17±2,76 for Group-I and 3,80±1,99 for Group-II (p < 0.05) (Table-I). Five patients (12.5%) were transfused in Group-I and two (3.57%) patients were transfused in Group-II (p=0.097). The post-PCNL fever rate was 10% in Group-I and 12.5% in Group-II (p=0.705).

**Table-I:** The general characteristics of the patients and comparison of two antibiotic groups.

Groups	Ciprofloxacin (Group I)			Cefuroxim (Group II)			t	z	χ	p

Parameter	Min-Max	Mean±SD	Median-IQR	Min-Max	Mean±SD	Median-IQR				
Age (year)	18-77	42,75±16,97	40-29	18-79	35,54±14,71	32,50-21		-2,201		0,028
BMI (kg/m^2^)	18-38	26,78±4,91	26-7	17-40	27,64±5,16	27-8	-0,829			0,409
Stone size (mm)	16-45	27,23±7,05	28-12	16-50	30,59±8,20	31,50-10	-2,098			0,039
Preoperative INR	0,8-1,20	0,99±0,11	0,97-0,20	0,80-1,20	0,98±0,09	0,98-0,11	4,046			0,59
Preoperative Plt count (10^3^/uL)	130-475	268,97±93,61	239-158	115-455	243,27±75,71	246-107		-1,160		0,246
Postoperative Plt count (10^3^/uL)	110-360	226,83±74,19	201,50-111	123-398	206,29±63,16	200-87		-1,227		0,220
Plt difference (10^3^/uL)	-225-25	-39,30±49,86	-39,50-73	-155-48	-35,38±53,05	-31-83		-0,316		0,752
Hb drop (g/dL)	0,4-4,10	1,73±0,95	1,50-1,15	0,3-4,1	1,28±0,67	1,10-0,70		-2,561		0,010
Hct drop	1,3-12,20	5,17±2,76	4,40-3,48	1,00-12,00	3,80±1,99	3,30-2,08		-2,762		0,006
Operation time (min)	55-150	98,48±27,12	108-49	61-155	103,09±26,78	100-47		-0,915		0,360
Postoperative stay (day)	2-11	3,43±1,68	3-1	2-10	3,21±1,50	3-1		-1,134		0,257

## DISCUSSION

PCNL is a popular treatment modality of prefer for many kidney stones larger than two cm, staghorn kidney stones and lower pole stones larger than 1cm. Although PCNL has a number of advantages such as shorter hospital stay, higher stone clearance, less pain, a quicker return to daily activities, smaller incision and cost-effectiveness compared with open surgery, it is associated with several complications such as bleeding necessitating blood transfusion, adjacent organ injury, collecting system perforation, postoperative renal colic, fever, urinary infection and septicemia. Among these complications, bleeding is the most common major complication of PCNL, necessitating blood transfusion, with an incidence reported between 0.8% and 45%.^4^ It can usually be managed conservatively except for arteriovenous fistula, pseudoaneurysm and massive bleeding. In these cases, endovascular interventional radiologic procedures, like as angioembolization, may be required. In 75 to 90% of all surgical procedures, intraoperative and early postoperative bleeding are associated with a technical defect.^5^,^6^ Intraoperative bleeding is the most commonly caused by structural defects, use of anticoagulant, hyperfibrinolysis or disorders of hemostasis such as disseminated intravascular coagulation. Early postoperative hemorrhage (within two days of surgery) indicates a defect in primary hemostasis, such as platelet dysfunction or significant thrombocytopenia, each of which may be acquired or inherited.^7^ It has been reported that many factors are associated with increased blood loss in PCNL procedures, including surgeon experience and technique, prolonged operation time, preoperative anemia, diabetes, patient age, high BMI, increased stone burden, need for multiple or larger access tracts and history of previous renal surgery.^8^,^9^ The literature review shows that there is no knowledge about the relation between increased blood loss and used antibiotics in the PCNL.

Surgical technique is a significant risk factor for intraoperative and postoperative bleeding in PCNL procedures. Mostly, percutaneous renal access should be performed through a posterior calyx along its axis to avoid blood vessels that course alongside the infundibulum.^10^ Renal tract for PCNL should be dilated to lateral side of the collecting system, as medial dilation increases the risk of renal pelvic injury.^9^ Sometimes, multiple accesses may be required for the removal of complex and staghorn calculi which also increase the risk of bleeding.^11^ It was reported that the risk of hemorrhage was increased 4.46 times more in multi-tract PCNL group compared with single-tract group.^4^ Whereas some studies support this idea, it was reported that the risk of hemorrhage was not increased in various studies.^11^-^13^ Access to the lower calyx stones is often easier and faster. For this reason, we included the patients who were performed only single posterior lower calyceal access in our study in order to evaluate the bleeding more objectively.

Prolonged operation time and increased intraoperative bleeding may cause a vicious circle. Increased bleeding during prolonged operations results in poor visualization during PCNL, which may further prolong the operative time and increases the amount of bleeding. A recent study of 2486 patients determined that the mean operation time was 86 ± 37 minutes.^14^ Akman et al. reported that the best cutoff point of operative time for necessitating blood transfusion is 58 minutes, and for operative times exceeding 58 minutes, blood transfusion requirement increased 2.82 times.^4^ In our study, the mean operation times were longer in the both antibiotic groups than in their study. We calculated the operative time as based on procedural start and stop times while they calculated the operative time as the time from the puncture until the final placement of the nephrostomy tube.

Stone size is considered as an important predictive factor for bleeding. Srivastava and colleagues reported that the stone size was the only significant factor that could predict the occurrence of blood loss in PCNL procedures.^15^ Akman et al. reported that the best cutoff point of stone size was 1250 mm^2^ for necessitating blood in staghorn calculi.^4^ Lee et al. reported that intraoperative and the postoperative bleeding in PCNL was not significantly different according to composition and radiopacity of stones.^3^ The composition of stones was not routinely analyzed in our cases. In our study, although the stone size was lower in the ciprofloxacin group than in the cefuroxime group, the decrease of hemoglobin and hematocrit was statistically significant higher. Need for postoperative blood transfusion was higher in the ciprofloxacin group than in the cefuroxime group, but it was not statistically significant.

Renal parenchymal bleeding can be managed by moving Amplatz sheath during the procedure. However, the tamponade is lost at the end of procedure. This tamponade effect is provided postoperatively by a nephrostomy tube. Jamil et al. reported that use of nephrostomy tube with balloon, such as Foley catheter, after PCNL as this is associated with less hemoglobin drop as compare to nephrostomy tube without balloon.^16^ We routinely used a 16F nephrostomy tube without balloon for this purpose.

Some antibiotics are known to have caused hemorrhage.^17^ Some antibiotics now being used have shown to cause a variety of coagulopathies including hemolytic anemia, prolonged bleeding time and thrombocytopenia.^18^ The evidences supporting the relationship between antibiotics and these coagulopathies are mainly based on case reports. Cefuroxime and ciprofloxacin are two commonly used antibiotics in urologic surgeries. Both antibiotics are usually known as a well-tolerated and safe drug. Side-effects of ciprofloxacin are mild and not life-threatening, significant side-effects being seen in <1% cases.^19^ Hematological side effects are generally mild and seen in only 0.9-1.8% of all cases.^20^,^21^ Reported hematological side effects include reversible leucopenia and asymptomatic thrombocytopenia or thrombocytosis.^22^ A rare hematological side-effect is transiently acquired Von Willebrand syndrome that reported in two cases.^23^ Asymptomatic prolongation of bleeding time and bone marrow depression were reported in some cases.^22^,^24^ Cefuroxime axetil, a β-lactam antibiotic, is a second-generation cephalosporin. It has been used widely for long years in clinical practice for the treatment of various bacterial infections in adult, adolescent and pediatric patients. In recent trials, the overall incidence of adverse events possibly or probably related to cefuroxime axetil treatment ranged from 2 to 49%, with most reporting an incidence of <20%. Many of these side effects were mild to moderate and reversible, and few side effects were serious.^25^ These serious side effects include prolonged bleeding time, thrombocytopenia, and hemolytic anemia.^18^

The studies about the antibiotic induced bleeding are insufficient and most of them are case-based. Almost all have focused on antibiotic-related thrombocytopenia. In the study with the intensive care patients, it has been reported that use of ciprofloxacin may contribute to lower platelet count compared with using cefuroxime.^26^ In our study, mean postoperative platelets drop were higher in the ciprofloxacin group than in the cefuroxime group, although it was not statistically significant. However, mean postoperative hemoglobin and hematocrit drop were statistically significantly higher in ciprofloxacin group than in cefuroxime group.

The reason for more postoperative bleeding in the ciprofloxacin group is unclear. Hypothetically, it may be thought that ciprofloxacin may cause more platelet decline. However, it should be investigated that ciprofloxacin may cause platelet dysfunction, hemolytic anemia, or may have influenced other clotting mechanisms such as coagulation factors or vitamin K metabolism, too.

### Limitations of the study

The number of patients included in the study was inadequate due to the abundance of exclusion criteria. The other shortcomings are that the other preoperative and postoperative factors that could cause bleeding could not be investigated, and confounding factors could not be analyzed, because of the retrospective nature of the study.

## CONCLUSION

Many predictive factors for bleeding have been previously described in PCNL procedures. At first glance, the results of this initial study suggest that the antibiotic preference may also be one of the bleeding factors during and after operation. Multicenter prospective randomized controlled trials need to be undertaken to confirm antibiotic related bleeding in PCNL procedures.

### Authors’ Contribution

**AA:** Study design, manuscript preparation, data collection and analysis, is responsible for integrity of research.

**CA & MU:** Drafting and revising of manuscript.

**AA & MT:** Review and final approval of manuscript.
